# 4-(Dimethyl­amino)­pyridinium tetra­chlorido(quinoline-2-carboxyl­ato-κ^2^ *N*,*O*)stannate(IV)

**DOI:** 10.1107/S1600536811031473

**Published:** 2011-08-11

**Authors:** Ezzatollah Najafi, Mostafa M. Amini, Seik Weng Ng

**Affiliations:** a Department of Chemistry, General Campus, Shahid Beheshti University, Tehran 1983963113, Iran; b Department of Chemistry, University of Malaya, 50603 Kuala Lumpur, Malaysia; c Chemistry Department, Faculty of Science, King Abdulaziz University, PO Box 80203 Jeddah, Saudi Arabia

## Abstract

In the title salt, (C_7_H_11_N_2_)[SnCl_4_(C_10_H_6_NO_2_)], the Sn^IV^ atom is chelated by the *N*,*O*-bidentate carboxyl­ate ions and four chloride ions, showing a distorted octa­hedral SnNOCl_4_ coordination. In the crystal, the cation and anion are linked by a pyridinium–carboxyl­ate N—H⋯O hydrogen bond.

## Related literature

For a related ammonium tetra­chlorido(pyridine-2-carboxyl­ato)stannate(IV), see: Najafi *et al.* (2011 ▶).
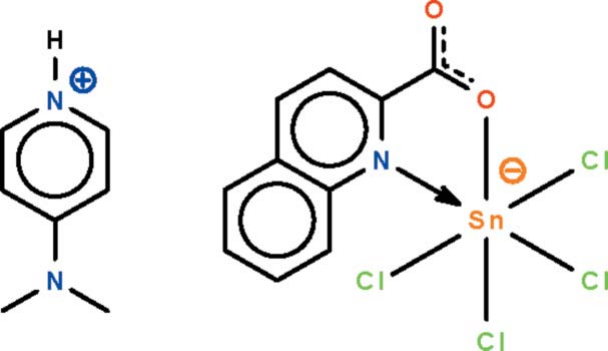


## Experimental

### 

#### Crystal data

(C_7_H_11_N_2_)[SnCl_4_(C_10_H_6_NO_2_)]
*M* *_r_* = 555.83
Triclinic, 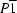

*a* = 8.6681 (3) Å
*b* = 8.8407 (4) Å
*c* = 14.4447 (5) Å
α = 96.721 (3)°
β = 91.924 (3)°
γ = 108.038 (4)°
*V* = 1042.43 (7) Å^3^
*Z* = 2
Mo *K*α radiation
μ = 1.76 mm^−1^
*T* = 100 K
0.30 × 0.20 × 0.10 mm

#### Data collection

Agilent SuperNova Dual diffractometer with an Atlas detector
Absorption correction: multi-scan (*CrysAlis PRO*; Agilent, 2010 ▶) *T*_min_ = 0.621, *T*_max_ = 0.844
8056 measured reflections
4610 independent reflections
4202 reflections with *I* > 2σ(*I*)
*R*_int_ = 0.022

#### Refinement

*R*[*F* ^2^ > 2σ(*F* ^2^)] = 0.021
*wR*(*F* ^2^) = 0.047
*S* = 1.06
4610 reflections
250 parameters
1 restraint
H atoms treated by a mixture of independent and constrained refinement
Δρ_max_ = 0.52 e Å^−3^
Δρ_min_ = −0.49 e Å^−3^

### 

Data collection: *CrysAlis PRO* (Agilent, 2010 ▶); cell refinement: *CrysAlis PRO*; data reduction: *CrysAlis PRO*; program(s) used to solve structure: *SHELXS97* (Sheldrick, 2008 ▶); program(s) used to refine structure: *SHELXL97* (Sheldrick, 2008 ▶); molecular graphics: *X-SEED* (Barbour, 2001 ▶); software used to prepare material for publication: *publCIF* (Westrip, 2010 ▶).

## Supplementary Material

Crystal structure: contains datablock(s) global, I. DOI: 10.1107/S1600536811031473/xu5284sup1.cif

Structure factors: contains datablock(s) I. DOI: 10.1107/S1600536811031473/xu5284Isup2.hkl

Additional supplementary materials:  crystallographic information; 3D view; checkCIF report

## Figures and Tables

**Table 1:** Hydrogen-bond geometry (Å, °)

*D*—H⋯*A*	*D*—H	H⋯*A*	*D*⋯*A*	*D*—H⋯*A*
N3—H3⋯O1	0.87 (1)	1.98 (1)	2.816 (2)	160 (2)

**Fig. 1.**
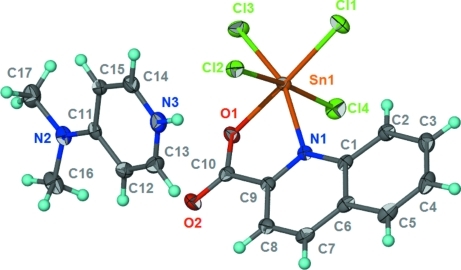
Thermal ellipsoid plot (Barbour, 2001) of (C7H11N2)[SnCl4(C10H6NO2)] at the 70% probability level; hydrogen atoms are drawn as spheres of arbitrary radius.

## supplementary crystallographic information

### Comment

We have recently synthesized some ammonium tetrachlorido(carboxylato)stannates; in a recent study, we reacted stannic chloride with pyridine-2-carboxylic acid and triethylamine to yield the chelated stannate salt (Najafi *et al.*, 2011). The use of quinoline-2-carboxylic acid and 4-dimethyaminopyridine yielded the expected dimethylaminopyridinium stannate in which the amine is protonated on the aromatic nitrogen atom (Scheme I, Fig. 1). The Sn^IV^ atom is chelated by the *N*,*O*-bidentate carboxylate ligand and four chloride ions, and shows octahedral SnNOCl_4_ coordination at the metal atom. The cation and anion are linked an N–H_pyridinium_···O hydrogen bond (Table 1).

### Experimental

Stannic chloride pentahydrate (1 mmol), quinoline-2-carboxylic acid (1 mmol) and 4-dimethylaminopyridine (1 mmol) were loaded into a convection tube and the tube was filled with dry methanol and kept at 333 K. Colorless crystals were collected from the side arm after several days.

### Refinement

Carbon-bound H-atoms were placed in calculated positions [C—H 0.95 to 0.98 Å, *U*_iso_(H) 1.2 to 1.5*U*_eq_(C)] and were included in the refinement in the riding model approximation.

The ammonium H-atom was located in a difference Fourier map, and was refined with a distance restraint of N–H 0.88±0.01 Å; its temperature factor was refined.

Omitted from the refinement was the (0 1 0) reflection.

### Crystal data

**Table:**

(C_7_H_11_N_2_)[SnCl_4_(C_10_H_6_NO_2_)]	*Z* = 2
*M**_r_* = 555.83	*F*(000) = 548
Triclinic, *P*1	*D*_x_ = 1.771 Mg m^−^^3^
Hall symbol: -P 1	Mo *K*α radiation, λ = 0.71073 Å
*a* = 8.6681 (3) Å	Cell parameters from 5833 reflections
*b* = 8.8407 (4) Å	θ = 2.4–29.2°
*c* = 14.4447 (5) Å	µ = 1.76 mm^−^^1^
α = 96.721 (3)°	*T* = 100 K
β = 91.924 (3)°	Block, colorless
γ = 108.038 (4)°	0.30 × 0.20 × 0.10 mm
*V* = 1042.43 (7) Å^3^	

### Data collection

**Table:**

Agilent SuperNova Dual diffractometer with an Atlas detector	4610 independent reflections
Radiation source: SuperNova (Mo) X-ray Source	4202 reflections with *I* > 2σ(*I*)
Mirror	*R*_int_ = 0.022
Detector resolution: 10.4041 pixels mm^-1^	θ_max_ = 27.5°, θ_min_ = 2.5°
ω scans	*h* = −8→11
Absorption correction: multi-scan (*CrysAlis PRO*; Agilent, 2010)	*k* = −11→11
*T*_min_ = 0.621, *T*_max_ = 0.844	*l* = −18→18
8056 measured reflections	

### Refinement

**Table:**

Refinement on *F*^2^	Primary atom site location: structure-invariant direct methods
Least-squares matrix: full	Secondary atom site location: difference Fourier map
*R*[*F*^2^ > 2σ(*F*^2^)] = 0.021	Hydrogen site location: inferred from neighbouring sites
*wR*(*F*^2^) = 0.047	H atoms treated by a mixture of independent and constrained refinement
*S* = 1.06	*w* = 1/[σ^2^(*F*_o_^2^) + (0.0181*P*)^2^ + 0.127*P*] where *P* = (*F*_o_^2^ + 2*F*_c_^2^)/3
4610 reflections	(Δ/σ)_max_ = 0.001
250 parameters	Δρ_max_ = 0.52 e Å^−^^3^
1 restraint	Δρ_min_ = −0.49 e Å^−^^3^

### Fractional atomic coordinates and isotropic or equivalent isotropic displacement parameters (Å^2^)

**Table:**

	*x*	*y*	*z*	*U*_iso_*/*U*_eq_	
Sn1	0.361135 (16)	0.258730 (15)	0.271428 (9)	0.01232 (5)	
Cl1	0.16903 (6)	0.37830 (6)	0.33361 (4)	0.02211 (12)	
Cl2	0.13953 (6)	0.04597 (6)	0.18599 (3)	0.01824 (11)	
Cl3	0.41373 (6)	0.41174 (6)	0.14341 (4)	0.02133 (11)	
Cl4	0.58090 (6)	0.44245 (6)	0.37084 (4)	0.02246 (12)	
O1	0.51697 (17)	0.14313 (16)	0.21142 (9)	0.0165 (3)	
O2	0.57573 (18)	−0.08559 (17)	0.19327 (10)	0.0207 (3)	
N1	0.36250 (19)	0.06808 (18)	0.36458 (10)	0.0124 (3)	
N2	1.0685 (2)	0.3405 (2)	−0.12106 (11)	0.0173 (4)	
N3	0.7058 (2)	0.2281 (2)	0.05939 (12)	0.0203 (4)	
H3	0.629 (2)	0.206 (3)	0.0979 (13)	0.024 (6)*	
C1	0.2921 (2)	0.0400 (2)	0.44784 (13)	0.0134 (4)	
C2	0.2404 (2)	0.1582 (2)	0.49972 (13)	0.0173 (4)	
H2	0.2535	0.2582	0.4777	0.021*	
C3	0.1712 (3)	0.1279 (3)	0.58221 (14)	0.0203 (5)	
H3A	0.1369	0.2080	0.6172	0.024*	
C4	0.1499 (3)	−0.0194 (3)	0.61614 (14)	0.0219 (5)	
H4	0.0987	−0.0389	0.6726	0.026*	
C5	0.2022 (2)	−0.1341 (3)	0.56843 (14)	0.0194 (5)	
H5	0.1888	−0.2327	0.5923	0.023*	
C6	0.2768 (2)	−0.1071 (2)	0.48305 (13)	0.0158 (4)	
C7	0.3379 (2)	−0.2201 (2)	0.43302 (14)	0.0172 (4)	
H7	0.3269	−0.3199	0.4550	0.021*	
C8	0.4130 (2)	−0.1860 (2)	0.35297 (13)	0.0164 (4)	
H8	0.4577	−0.2598	0.3196	0.020*	
C9	0.4230 (2)	−0.0400 (2)	0.32101 (13)	0.0133 (4)	
C10	0.5114 (2)	0.0037 (2)	0.23433 (13)	0.0145 (4)	
C11	0.9504 (2)	0.3054 (2)	−0.06169 (13)	0.0143 (4)	
C12	0.9714 (3)	0.2416 (2)	0.02178 (14)	0.0184 (4)	
H12	1.0717	0.2248	0.0375	0.022*	
C13	0.8486 (3)	0.2047 (2)	0.07897 (14)	0.0205 (5)	
H13	0.8638	0.1612	0.1344	0.025*	
C14	0.6811 (3)	0.2898 (2)	−0.01880 (14)	0.0199 (5)	
H14	0.5797	0.3062	−0.0316	0.024*	
C15	0.7983 (2)	0.3288 (2)	−0.07943 (14)	0.0166 (4)	
H15	0.7784	0.3720	−0.1341	0.020*	
C16	1.2127 (3)	0.2906 (3)	−0.10898 (16)	0.0242 (5)	
H16A	1.2761	0.3488	−0.0511	0.036*	
H16B	1.2794	0.3146	−0.1621	0.036*	
H16C	1.1793	0.1750	−0.1057	0.036*	
C17	1.0413 (3)	0.3978 (3)	−0.20898 (14)	0.0258 (5)	
H17A	0.9928	0.4840	−0.1971	0.039*	
H17B	0.9676	0.3091	−0.2522	0.039*	
H17C	1.1454	0.4388	−0.2367	0.039*	

### Atomic displacement parameters (Å^2^)

**Table:**

	*U*^11^	*U*^22^	*U*^33^	*U*^12^	*U*^13^	*U*^23^
Sn1	0.01240 (7)	0.01035 (8)	0.01461 (8)	0.00357 (6)	0.00258 (5)	0.00280 (5)
Cl1	0.0238 (3)	0.0179 (3)	0.0275 (3)	0.0104 (2)	0.0071 (2)	0.0023 (2)
Cl2	0.0183 (3)	0.0146 (2)	0.0197 (2)	0.0034 (2)	−0.0038 (2)	0.00033 (19)
Cl3	0.0229 (3)	0.0214 (3)	0.0235 (3)	0.0086 (2)	0.0061 (2)	0.0125 (2)
Cl4	0.0208 (3)	0.0168 (3)	0.0243 (3)	−0.0010 (2)	−0.0032 (2)	0.0012 (2)
O1	0.0185 (7)	0.0168 (7)	0.0173 (7)	0.0084 (6)	0.0074 (6)	0.0045 (6)
O2	0.0244 (8)	0.0229 (8)	0.0197 (8)	0.0145 (7)	0.0054 (6)	0.0025 (6)
N1	0.0110 (8)	0.0117 (8)	0.0134 (8)	0.0021 (7)	0.0000 (7)	0.0015 (6)
N2	0.0155 (9)	0.0163 (9)	0.0181 (9)	0.0026 (7)	0.0042 (7)	0.0005 (7)
N3	0.0200 (10)	0.0245 (10)	0.0175 (9)	0.0072 (8)	0.0089 (8)	0.0042 (7)
C1	0.0110 (9)	0.0149 (10)	0.0122 (9)	0.0010 (8)	−0.0007 (8)	0.0027 (8)
C2	0.0167 (10)	0.0170 (11)	0.0171 (10)	0.0039 (9)	−0.0004 (8)	0.0024 (8)
C3	0.0164 (11)	0.0256 (12)	0.0177 (10)	0.0062 (9)	0.0013 (9)	−0.0013 (9)
C4	0.0159 (11)	0.0329 (13)	0.0134 (10)	0.0027 (10)	0.0022 (8)	0.0030 (9)
C5	0.0159 (10)	0.0194 (11)	0.0183 (10)	−0.0027 (9)	−0.0010 (9)	0.0075 (9)
C6	0.0126 (10)	0.0164 (10)	0.0155 (10)	0.0006 (8)	−0.0029 (8)	0.0032 (8)
C7	0.0187 (11)	0.0117 (10)	0.0187 (10)	0.0012 (9)	−0.0048 (9)	0.0034 (8)
C8	0.0166 (10)	0.0146 (10)	0.0169 (10)	0.0052 (9)	−0.0034 (8)	−0.0013 (8)
C9	0.0113 (9)	0.0139 (10)	0.0136 (10)	0.0033 (8)	−0.0012 (8)	0.0003 (8)
C10	0.0120 (10)	0.0171 (10)	0.0136 (10)	0.0039 (8)	−0.0016 (8)	0.0019 (8)
C11	0.0157 (10)	0.0098 (9)	0.0154 (10)	0.0021 (8)	0.0023 (8)	−0.0022 (7)
C12	0.0170 (10)	0.0204 (11)	0.0193 (10)	0.0084 (9)	0.0006 (9)	0.0020 (8)
C13	0.0245 (12)	0.0204 (11)	0.0179 (10)	0.0077 (10)	0.0017 (9)	0.0062 (8)
C14	0.0178 (11)	0.0230 (12)	0.0198 (11)	0.0096 (9)	0.0000 (9)	−0.0020 (9)
C15	0.0195 (11)	0.0174 (10)	0.0143 (10)	0.0086 (9)	−0.0002 (8)	0.0002 (8)
C16	0.0132 (10)	0.0261 (12)	0.0317 (12)	0.0054 (9)	0.0070 (9)	−0.0015 (10)
C17	0.0333 (13)	0.0236 (12)	0.0204 (11)	0.0065 (10)	0.0118 (10)	0.0068 (9)

### Geometric parameters (Å, °)

**Table:**

Sn1—O1	2.0848 (13)	C4—H4	0.9500
Sn1—N1	2.2790 (16)	C5—C6	1.422 (3)
Sn1—Cl1	2.3802 (5)	C5—H5	0.9500
Sn1—Cl4	2.3840 (5)	C6—C7	1.409 (3)
Sn1—Cl3	2.3912 (5)	C7—C8	1.365 (3)
Sn1—Cl2	2.4106 (5)	C7—H7	0.9500
O1—C10	1.301 (2)	C8—C9	1.400 (3)
O2—C10	1.213 (2)	C8—H8	0.9500
N1—C9	1.333 (2)	C9—C10	1.516 (3)
N1—C1	1.381 (2)	C11—C15	1.416 (3)
N2—C11	1.343 (2)	C11—C12	1.419 (3)
N2—C17	1.459 (3)	C12—C13	1.353 (3)
N2—C16	1.460 (3)	C12—H12	0.9500
N3—C13	1.343 (3)	C13—H13	0.9500
N3—C14	1.348 (3)	C14—C15	1.353 (3)
N3—H3	0.869 (9)	C14—H14	0.9500
C1—C2	1.408 (3)	C15—H15	0.9500
C1—C6	1.421 (3)	C16—H16A	0.9800
C2—C3	1.369 (3)	C16—H16B	0.9800
C2—H2	0.9500	C16—H16C	0.9800
C3—C4	1.406 (3)	C17—H17A	0.9800
C3—H3A	0.9500	C17—H17B	0.9800
C4—C5	1.360 (3)	C17—H17C	0.9800
			
O1—Sn1—N1	75.24 (5)	C7—C6—C5	122.27 (19)
O1—Sn1—Cl1	176.27 (4)	C1—C6—C5	118.72 (19)
N1—Sn1—Cl1	104.74 (4)	C8—C7—C6	119.87 (18)
O1—Sn1—Cl4	90.93 (4)	C8—C7—H7	120.1
N1—Sn1—Cl4	88.46 (4)	C6—C7—H7	120.1
Cl1—Sn1—Cl4	92.797 (19)	C7—C8—C9	118.64 (19)
O1—Sn1—Cl3	85.08 (4)	C7—C8—H8	120.7
N1—Sn1—Cl3	160.21 (4)	C9—C8—H8	120.7
Cl1—Sn1—Cl3	94.762 (18)	N1—C9—C8	123.44 (17)
Cl4—Sn1—Cl3	93.991 (19)	N1—C9—C10	116.83 (17)
O1—Sn1—Cl2	87.29 (4)	C8—C9—C10	119.70 (17)
N1—Sn1—Cl2	83.40 (4)	O2—C10—O1	124.30 (18)
Cl1—Sn1—Cl2	89.002 (18)	O2—C10—C9	120.59 (18)
Cl4—Sn1—Cl2	171.852 (17)	O1—C10—C9	115.05 (17)
Cl3—Sn1—Cl2	93.779 (18)	N2—C11—C15	121.88 (18)
C10—O1—Sn1	118.81 (12)	N2—C11—C12	121.52 (19)
C9—N1—C1	119.22 (16)	C15—C11—C12	116.60 (18)
C9—N1—Sn1	110.22 (12)	C13—C12—C11	119.88 (19)
C1—N1—Sn1	129.82 (13)	C13—C12—H12	120.1
C11—N2—C17	120.69 (18)	C11—C12—H12	120.1
C11—N2—C16	120.42 (17)	N3—C13—C12	121.67 (19)
C17—N2—C16	117.65 (17)	N3—C13—H13	119.2
C13—N3—C14	120.35 (18)	C12—C13—H13	119.2
C13—N3—H3	120.3 (15)	N3—C14—C15	121.2 (2)
C14—N3—H3	119.4 (15)	N3—C14—H14	119.4
N1—C1—C2	120.50 (17)	C15—C14—H14	119.4
N1—C1—C6	119.72 (17)	C14—C15—C11	120.29 (19)
C2—C1—C6	119.74 (17)	C14—C15—H15	119.9
C3—C2—C1	119.50 (19)	C11—C15—H15	119.9
C3—C2—H2	120.2	N2—C16—H16A	109.5
C1—C2—H2	120.2	N2—C16—H16B	109.5
C2—C3—C4	121.3 (2)	H16A—C16—H16B	109.5
C2—C3—H3A	119.3	N2—C16—H16C	109.5
C4—C3—H3A	119.3	H16A—C16—H16C	109.5
C5—C4—C3	120.29 (19)	H16B—C16—H16C	109.5
C5—C4—H4	119.9	N2—C17—H17A	109.5
C3—C4—H4	119.9	N2—C17—H17B	109.5
C4—C5—C6	120.34 (19)	H17A—C17—H17B	109.5
C4—C5—H5	119.8	N2—C17—H17C	109.5
C6—C5—H5	119.8	H17A—C17—H17C	109.5
C7—C6—C1	119.00 (17)	H17B—C17—H17C	109.5
			
N1—Sn1—O1—C10	17.16 (14)	C4—C5—C6—C1	−1.4 (3)
Cl4—Sn1—O1—C10	105.32 (14)	C1—C6—C7—C8	1.2 (3)
Cl3—Sn1—O1—C10	−160.75 (14)	C5—C6—C7—C8	−177.90 (19)
Cl2—Sn1—O1—C10	−66.72 (14)	C6—C7—C8—C9	−1.9 (3)
O1—Sn1—N1—C9	−16.03 (12)	C1—N1—C9—C8	2.9 (3)
Cl1—Sn1—N1—C9	160.11 (12)	Sn1—N1—C9—C8	−168.24 (16)
Cl4—Sn1—N1—C9	−107.40 (12)	C1—N1—C9—C10	−174.97 (16)
Cl3—Sn1—N1—C9	−9.9 (2)	Sn1—N1—C9—C10	13.9 (2)
Cl2—Sn1—N1—C9	72.90 (12)	C7—C8—C9—N1	−0.2 (3)
O1—Sn1—N1—C1	174.03 (17)	C7—C8—C9—C10	177.68 (17)
Cl1—Sn1—N1—C1	−9.83 (16)	Sn1—O1—C10—O2	167.37 (15)
Cl4—Sn1—N1—C1	82.65 (16)	Sn1—O1—C10—C9	−15.3 (2)
Cl3—Sn1—N1—C1	−179.84 (11)	N1—C9—C10—O2	176.83 (18)
Cl2—Sn1—N1—C1	−97.05 (16)	C8—C9—C10—O2	−1.1 (3)
C9—N1—C1—C2	174.22 (18)	N1—C9—C10—O1	−0.6 (3)
Sn1—N1—C1—C2	−16.6 (3)	C8—C9—C10—O1	−178.61 (17)
C9—N1—C1—C6	−3.6 (3)	C17—N2—C11—C15	−2.8 (3)
Sn1—N1—C1—C6	165.62 (14)	C16—N2—C11—C15	−169.76 (18)
N1—C1—C2—C3	−179.86 (18)	C17—N2—C11—C12	176.65 (18)
C6—C1—C2—C3	−2.1 (3)	C16—N2—C11—C12	9.6 (3)
C1—C2—C3—C4	−0.3 (3)	N2—C11—C12—C13	−178.67 (19)
C2—C3—C4—C5	1.8 (3)	C15—C11—C12—C13	0.8 (3)
C3—C4—C5—C6	−0.9 (3)	C14—N3—C13—C12	0.0 (3)
N1—C1—C6—C7	1.6 (3)	C11—C12—C13—N3	−0.5 (3)
C2—C1—C6—C7	−176.23 (18)	C13—N3—C14—C15	0.3 (3)
N1—C1—C6—C5	−179.32 (17)	N3—C14—C15—C11	−0.1 (3)
C2—C1—C6—C5	2.9 (3)	N2—C11—C15—C14	178.95 (19)
C4—C5—C6—C7	177.70 (19)	C12—C11—C15—C14	−0.5 (3)

### Hydrogen-bond geometry (Å, °)

**Table:**

*D*—H···*A*	*D*—H	H···*A*	*D*···*A*	*D*—H···*A*
N3—H3···O1	0.87 (1)	1.98 (1)	2.816 (2)	160 (2)
